# Compared with Daily, Weekly n–3 PUFA Intake Affects the Incorporation of Eicosapentaenoic Acid and Docosahexaenoic Acid into Platelets and Mononuclear Cells in Humans^1^,^2^,^3^

**DOI:** 10.3945/jn.113.186346

**Published:** 2014-03-19

**Authors:** Lucy M. Browning, Celia G. Walker, Adrian P. Mander, Annette L. West, Joanna Gambell, Jackie Madden, Philip C. Calder, Susan A. Jebb

**Affiliations:** 4 Medical Research Council Human Nutrition Research, Cambridge, United Kingdom; 5 Medical Research Council Biostatistics Unit Hub for Trials Methodology Research, Institute of Public Health, University Forvie Site, Cambridge, United Kingdom; 6 Human Development and Health Academic Unit, Faculty of Medicine, University of Southampton, Southampton, United Kingdom; and; 7 National Institute of Health Research Southampton Biomedical Research Centre, University of Southampton and University Hospital Southampton National Health Service Foundation Trust, Southampton, United Kingdom

## Abstract

Consumption of oily fish is sporadic, whereas controlled intervention studies of n–3 (ω-3) fatty acids usually provide capsules containing eicosapentaenoic acid (EPA) and docosahexaenoic acid (DHA) as a daily dose. This methodologic study explored whether there are differences in the short-, medium-, and long-term incorporation of EPA and DHA into blood plasma and cells with the provision of identical amounts of EPA and DHA, equivalent to 2 oily fish servings per week (or 6.54 g/wk EPA and DHA), either intermittently (i.e., 1 portion twice per week) or continuously (i.e., divided into daily amounts). The study was part of a randomized, double-blind controlled intervention lasting 12 mo, with participants stratified by age and sex. There were 5 intervention groups, 2 of which are reported here: the 2 intermittent portions (2I) and 2 continuous portions (2C) groups. EPA and DHA were measured in plasma phosphatidylcholine, platelets, and blood mononuclear cells (MNCs) at 9 time points. Sixty-five participants completed the study (2I group, *n* = 30, mean age of 49.2 y; 2C group, *n* = 35, mean age of 50.6 y). The incorporation pattern over the 12-mo intervention was different between the 2 groups in all samples (*P* < 0.0001, time × treatment interaction). At the end of the 12-mo intervention, the 2C group had higher EPA, DHA, and EPA + DHA in platelets (all *P* < 0.01) and higher EPA and EPA + DHA in MNCs (both *P* < 0.05) compared with the 2I group. No significant differences were shown for plasma phosphatidylcholine EPA (*P* = 0.1), DHA (*P* = 0.15), EPA + DHA (*P* = 0.07), or MNC DHA (*P* = 0.06). In conclusion, the pattern of consumption does affect the incorporation of EPA and DHA into cells used as biomarkers of intake. The differences identified here need to be considered in the design of studies and when extrapolating results from continuous capsule-based intervention studies to dietary guidelines for oily fish consumption. This trial was registered at www.controlled-trials.com as ISRCTN48398526.

## Introduction

There is good epidemiologic evidence to support the positive effect of the consumption of oily fish in reducing risk of disease, including all-cause mortality, cardiovascular disease (1–4), stroke (5), and diabetes (6, 7). There is mounting evidence from intervention studies to increase dietary oily fish consumption to beneficially alter risk factors of disease (8–10). The benefit of fish consumption is considered to lie with its component n–3 PUFAs, EPA and DHA. Indeed, the balance of evidence from intervention studies uses marine-derived n–3 PUFAs (i.e., EPA and DHA) as oral supplements, which shows reductions in various disease risk factors (11–13). Although it is assumed that exposure to EPA and DHA delivered either in supplement form or as oily fish will induce the same biologic effects, there are potential differences in both impact of the pattern of consumption of n–3 PUFAs from food and supplements and the overall pattern and composition of a diet containing oily fish compared with one supplemented with capsules.

The provision of EPA and DHA during supplementation studies differs from that of dietary intake, with oily fish being consumed sporadically in most populations. In the United Kingdom, dietary intake data from the National Diet and Nutrition Survey show that, on average, <1 meal containing oily fish is eaten each week, and target dietary guidelines recommend 1–2 portions of oily fish per week (14). In contrast, supplements providing EPA and DHA are typically taken daily. This difference in the pattern of delivery of EPA and DHA may alter the uptake and enrichment of EPA and DHA into the blood, cells, and tissues. Most of the evidence on which dietary recommendations are based is derived from supplementation studies, yet a difference in uptake and incorporation could have implications for the interpretation of these data and the subsequent translation to public health advice, which is usually given in terms of portions of oily fish per week.

The aim of this study was to determine whether there is a difference in EPA and DHA measured in biomarkers of intake when given sporadically compared with a regular amount consumed every day, at doses that reflect typical dietary oily fish intake. This study forms a secondary but prespecified aim of a larger study. The primary outcome of the full study, to elucidate the dose and time response of enrichment of EPA and DHA in plasma fractions, cells, and adipose tissue with intermittent supplementation, has been reported previously (15). We identified that plasma phosphatidylcholine EPA and DHA was the best marker of short-term changes in intake, platelets as a medium-term marker of intake, and blood mononuclear cells (MNCs)^8^ as a long-term marker of intake (15). Although we also collected other sample types (plasma cholesteryl esters, plasma non-esterified FAs, plasma TGs, erythrocyte membranes, buccal cells, and adipose tissue), we do not report their results here.

## Materials and Methods

### 

#### Study design, participants, and ethics statement.

**FIGURE 1:**
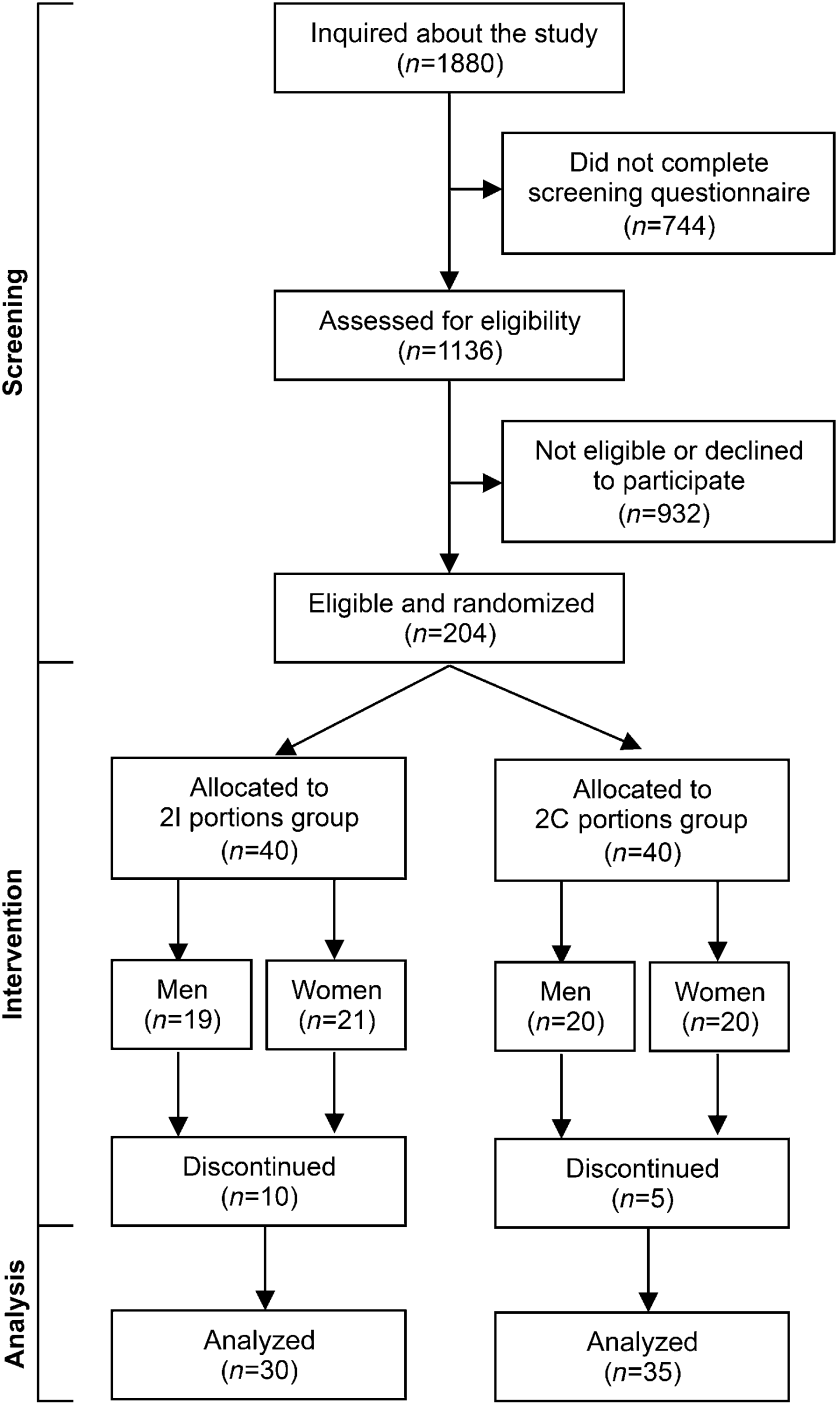
Consort flow diagram of participants receiving intermittent (2I group) or continuous (2C group) n–3 PUFA supplementation during a 12-mo intervention in men and women. The consort diagram for the full trial, for which the results of the other intervention groups have been reported previously (15), is shown in Supplemental Figure 1, including details for participants who were not eligible or declined to participate. 2C, 2 continuous portions; 2I, 2 intermittent portions.

This study forms part of a larger study, the design and methods of which have been described previously in detail (15). Briefly, a double-blind, randomized, controlled intervention trial was conducted with 5 parallel groups, set in 2 centers within the United Kingdom and lasting 12 mo (**Supplemental Fig. 1**). Two of the 5 parallel groups are described here (**Fig. 1**). The study was registered at www.controlled-trials.com as ISRCTN48398526. All procedures were approved by the Suffolk Local Research Ethics Committee (approval 05/Q0102/181), and written informed consent was obtained by all participants. Participants who reported no habitual consumption of oily fish (defined as <1 portion/mo) were recruited in Cambridge and Southampton, United Kingdom, and random assignment was stratified by age (“young”: 20–39 y; “middle”: 40–59 y; and “old”: 60–79 y) and sex (male, female). Participants were seen at 9 study time points: *1*) baseline; *2*) 1 wk; *3*) 2 wk; *4*) 1 mo; *5*) 2 mo; *6*) 3 mo; *7*) 6 mo; *8*) 9 mo; and *9*) 12 mo. Exclusion criteria were as follows: *1*) diagnosed diabetes, cancer, cardiovascular disease, or other chronic clinical conditions; *2*) untreated hypertension; *3*) concomitant prescription of anticoagulants, non-steroidal anti-inflammatory drugs, aspirin, steroids, or immunosuppressants; *4*) allergy or intolerance to fish; *5*) consumption of fish oil supplements in the past 3 mo; *6*) consumption of oily fish more than once a month; *7*) smoking; *8*) history of substance abuse or alcoholism; *9*) pregnant, <1 y postpartum, or planning pregnancy; *10*) recent weight change (>2 kg in the past 1 mo) or planning to change dietary habits, increase physical activity, or change body weight; *11*) planning to move away from the study center locality or take a lengthy vacation during the time of the study; and *12*) BMI <18 or >35 kg/m^2^.

#### Intervention.

**FIGURE 2:**
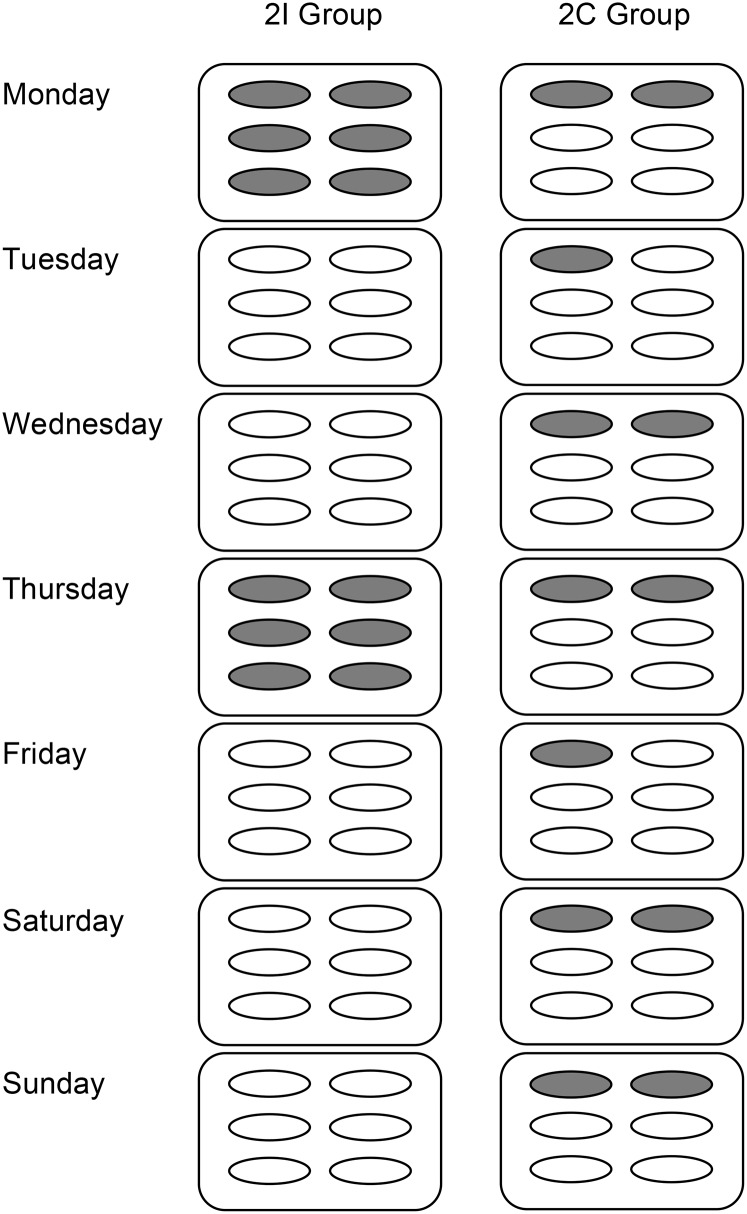
Design of the individual day blister packs, providing EPA and DHA equivalent to 2 portions of oily fish per week in the 2I and 2C groups in a 12-mo intervention of intermittent or continuous n–3 PUFA supplementation in men and women. We define 1 portion as 3.27 g of EPA and DHA, and 2 portions provides 3 g of EPA and 3.54 g of DHA, for a total of 6.54 g of EPA and DHA. Gray capsules indicate EPA and DHA capsules (providing 0.25 g of EPA and 0.30 g of DHA per capsule), and white capsules indicate placebo (high oleic sunflower oil, providing 0.58 g of oleic acid, 0.1 g of linoleic acid, 0.03 g of palmitic acid, and 0.02 g of stearic acid per capsule). The composition is detailed in a previously published online table (15). 2C, 2 continuous portions; 2I, 2 intermittent portions.

The intervention was capsule based, and all participants were administered 6 0.75-g capsules/d, as individual day blister packs, labeled with the day of the week. An average UK oily fish portion typically provides 2.8 g of EPA and DHA, and a typical portion of salmon provides 3.5 g of EPA and DHA (14). In the 2 intervention groups we describe, participants took a total of 6.54 g of EPA and DHA (3.0 g of EPA and 3.54 g of DHA) as TG per week (15), which we define as 2 portions of oily fish per week. The participants either took this dose of n–3 PUFAs on 2 of the 7 d of the week to simulate habitual consumption patterns [2 intermittent portions (2I) group] or distributed evenly over the 7 d [2 continuous portions (2C) group] (**Fig. 2**). Placebo oil capsules (high oleic acid sunflower oil) were given to make a total of 6 capsules/d and ensure blinding. Compliance was monitored at each study visit by return of the capsule packaging.

#### Dietary assessment.

To monitor for any changes in background diet, participants completed 3 unweighed 4-d diet diaries during the intervention (baseline, 6 mo, and 12 mo). Data were analyzed using an in-house dietary assessment system, “Diet in, Nutrients Out” (Medical Research Council Human Nutrition Research), that includes detailed data based on *McCance and Widdowson’s Composition of Foods Series* (16), Food Standards Agency *Food Portion Sizes* (17), and data from the manufacturers when applicable. At each of the 9 clinic visits, participants were specifically asked about consumption of oily and white fish since their previous visit.

#### Sample preparation and FA composition analysis.

Fasting blood samples were collected at each of the 9 clinic visits during the 12-mo intervention, from which plasma phosphatidylcholine, platelets, and MNCs were isolated and analyzed for FA composition. The preparation and analysis of blood samples has been described previously (15). Briefly, FAs were analyzed by GC, performed on a Hewlett Packard 6890 gas chromatograph fitted with a BPX-70 column (30 m × 0.22 mm × 0.25 *μ*m). The instrument was controlled by and data were collected using HPChemStation (Hewlett Packard). FAMEs were identified by comparison of retention times with those of authentic standards run previously.

#### Statistics.

Data were analyzed for participants completing the 12-mo intervention. For FA data, primary comparisons between all 5 intervention groups were analyzed using a repeated-measures mixed-effects model with the fixed factors treatment group, categorical time, treatment × time interactions, age (with levels young, middle, and old), sex (male vs. female), study center, baseline BMI, and average compliance over the 12 mo (percentage) and the random factor participant identification. Treatment × time interactions between the groups were performed by Wald test statistics on the model variables. A χ^2^ likelihood ratio test was used to test for treatment effect and report data for the 2I and 2C groups at 12 mo. Dietary data were analyzed as change from baseline to 12 mo and compared between groups using a *t* test. All analyses were performed with Stata version 12 (StataCorp).

## Results

### 

#### Participant characteristics.

The study design and flow of all participants in the original study are shown in Supplemental Figure 1, and those described in this publication are shown in Figure 1. Of the 204 participants in the original trial, 80 participants were enrolled into the 2 groups reported here (*n* = 40 per group). A total of 163 participants completed the 12 mo of intervention, with 65 participants reported in this analysis (*n* = 30 in the 2I group and *n* = 35 in the 2C group). The participant characteristics of the 2 groups at baseline are shown in **Table 1**. Analysis of baseline FA status in plasma phosphatidylcholine, MNCs, and platelets showed no significant differences in the proportion of EPA or DHA between the 2I and 2C groups (Table 1).

**TABLE 1:** Characteristics of the participants at baseline participating in a 12-mo intervention of intermittent or continuous n–3 PUFA supplementation in men and women^1^

	2I group	2C group
Gender, *n*		
Male	19	20
Female	21	20
Age, *y*	49.2 ± 14.9	50.6 ± 15.8
Weight, *kg*	75.1 ± 14.0	75.9 ± 16.0
BMI, *kg/m^2^*	25.5 ± 4.1	26.1 ± 3.9
Body fat, *%*	22.0 ± 9.0	22.4 ± 8.8
Systolic blood pressure, *mm Hg*	125 ± 16	126 ± 16
Diastolic blood pressure, *mm Hg*	73 ± 8	75 ± 8
Baseline plasma PC, *% total FA*		
EPA	1.17 ± 0.84	1.16 ± 0.54
DHA	3.69 ± 1.41	3.81 ± 1.32
Baseline platelets, *% total FA*		
EPA	1.15 ± 0.48	1.07 ± 0.51
DHA	2.18 ± 0.69	2.08 ± 0.53
Baseline MNCs, *% total FA*		
EPA	0.74 ± 0.49	0.72 ± 0.57
DHA	1.91 ± 0.52	1.91 ± 0.52

1 Values are means ± SDs or *n*. MNC, blood mononuclear cell; PC, phosphatidylcholine; 2C, 2 continuous portions; 2I, 2 intermittent portions.

Compliance assessed by returned capsule counting was high in both groups throughout the 12-mo intervention. Over the 12-mo intervention, mean values were 98.26% (IQR: 2.29) in the 2C group and 98.34% (IQR: 1.91) in the 2I group, with no significant difference in compliance between the groups.

#### Dietary intake.

Reported baseline total dietary n–3 PUFA intake in the 2I and 2C groups was lower than the UK national mean (2.2 ± 1.3 g/d), reflecting the recruitment of non-consumers of oily fish to the study. Data from 4-d diet diaries showed no significant differences for total fat, carbohydrate, or protein intake between the 2I and 2C groups at baseline or after the 12-mo intervention (**Table 2**). However, there was a small but statistically significant difference in the change in reported n–6 PUFA intake between the 2 groups (reflected also in the total PUFA measure), with an increase in the 2I group and a decrease in the 2C group.

**TABLE 2:** Reported dietary intake of participants during a 12-mo intervention of intermittent or continuous n–3 PUFA supplementation in men and women^1^

	Baseline		Change	
	2I group (*n* = 29)	2C group (*n* = 34)	2I group (*n* = 26)	2C group (*n* = 33)
	*% energy*		*% energy*	
Protein	16.4 ± 2.92	15.9 ± 2.60	0.34 ± 3.45	−0.49 ± 2.08
Carbohydrate	49.5 ± 8.46	47.0 ± 9.23	0.00 ± 8.23	0.16 ± 5.93
Total fat	33.9 ± 7.29	34.4 ± 6.16	0.51 ± 7.18	−0.56 ± 6.14
Total SFA	13.0 ± 3.53	12.6 ± 3.08	−0.35 ± 3.46	−0.02 ± 3.34
Total MUFA	11.4 ± 2.92	11.7 ± 2.08	−0.05 ± 3.22	−0.01 ± 2.29
Total PUFA	5.4 ± 1.78	5.9 ± 1.42	0.83 ± 1.79	−0.24 ± 1.89*
Total n–6 PUFA	4.7 ± 1.54	5.2 ± 1.31	0.86 ± 1.72	−0.24 ± 1.79*
Total n–3 PUFA	0.7 ± 0.31	0.8 ± 0.19	0.02 ± 0.30	−0.06 ± 0.24

1 Values are means ± SDs expressed as a percentage of total dietary energy per day. The *n* available for each group was dependent on the number of diaries completed and returned and is indicated in each column. *Different from the 2I group, *P* < 0.05. 2C, 2 continuous portions; 2I, 2 intermittent portions.

#### EPA and DHA content of short-, medium-, and long-term biomarkers of oily fish intake after 12-mo intervention.

**FIGURE 3:**
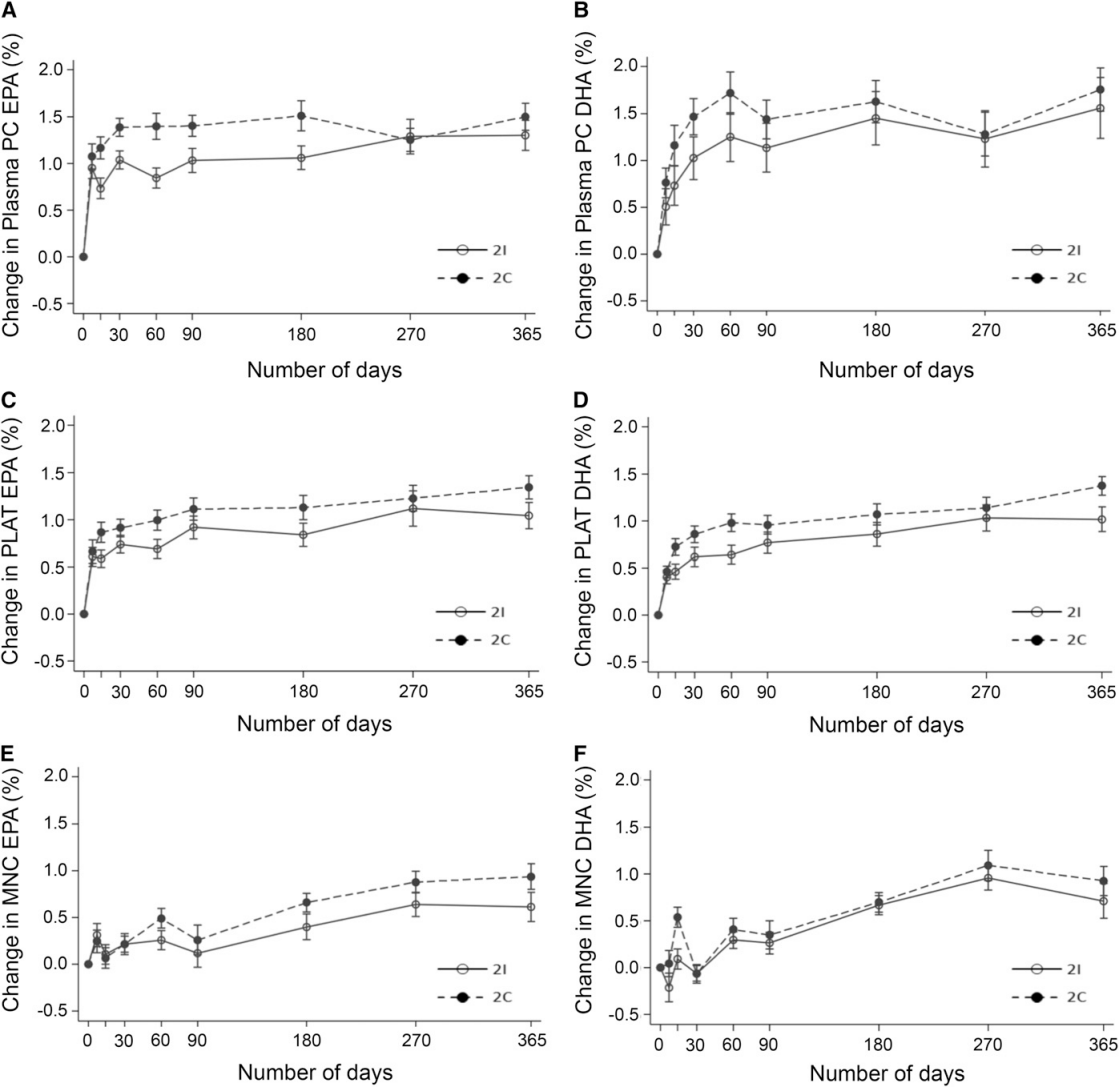
Changes in EPA (*A*, *C*, *E*) and DHA (*B*, *D*, *F*) in plasma PC, platelets, and MNCs during a 12-mo intervention of intermittent or continuous n–3 PUFA supplementation in men and women. Values are mean ± SE changes from baseline of EPA and DHA amounts (percentage of total FAs) over the 12-mo intervention providing EPA (3.0 g) and DHA (3.54 g) per week, equivalent to 2 oily fish portions per week. The number of plasma PC, platelet, and MNC samples available for analysis varied by time point and were in the range of *n* = 30–39 (2I group) and *n* = 33–40 (2C group). The differences are shown in (*A*) and (*B*) for plasma PC, (*C*) and (*D*) for platelets, and (*E*) and (*F*) for MNCs. MNC, blood mononuclear cell; PC, phosphatidylcholine; PLAT, platelet; 2C, 2 continuous portions; 2I, 2 intermittent portions.

The repeated-measures mixed-effects model showed a significant time × treatment interaction between the 2I and 2C groups for EPA, DHA, and EPA + DHA in plasma phosphatidylcholine, platelets, and MNCs (all *P* < 0.0001). The changes in EPA and DHA in plasma phosphatidylcholine, platelets, and MNC in the 2I and 2C groups over the 12-mo study are shown in **Figure 3**.

The mean amounts of EPA, DHA, and EPA + DHA as a percentage of total FAs at 12 mo in plasma phosphatidylcholine, platelets, and MNCs are shown in **Table 3**. The 2C group had significantly higher amounts of EPA, DHA, and EPA + DHA in platelets and of EPA and EPA + DHA in MNCs at 12 mo than the 2I group. There was no significant difference between the 2I or 2C groups in the mean amounts of EPA (*P* = 0.10), DHA (*P* = 0.15), or EPA + DHA (*P* = 0.07) in plasma phosphatidylcholine or of DHA in MNCs (*P* = 0.06) at 12 mo (Table 3). Additional adjustment of the models presented in Table 3 for change in dietary n–6 PUFA intake did not affect the significance of the results (data not shown).

**TABLE 3:** Differences in EPA, DHA, and EPA + DHA amounts at 12 mo between participants receiving intermittent or continuous n–3 PUFA supplementation in short-, medium-, and long-term biomarkers of oily fish intake^1^

	2I group^2^ (*n* = 30)	2C group^2^ (*n* = 35)	2I vs. 2C^3^	*P*
EPA				
Plasma PC	2.36 ± 0.75	2.67 ± 0.75	−0.28 (−0.61, 0.06)	0.10
Platelets	2.09 ± 0.40	2.51 ± 0.40	−0.40 (−0.70, −0.10)	0.009
MNCs	1.37 ± 0.25	1.66 ± 0.25	−0.29 (−0.55, −0.04)	0.02
DHA				
Plasma PC	5.19 ± 1.34	5.60 ± 1.34	−0.33 (−0.78, 0.12)	0.15
Platelets	3.13 ± 0.56	3.57 ± 0.56	−0.40 (−0.64, −0.16)	0.001
MNCs	2.57 ± 0.51	2.89 ± 0.51	−0.27 (−0.56, 0.01)	0.06
EPA + DHA				
Plasma PC	7.54 ± 2.00	8.27 ± 2.00	−0.63 (−1.29, 0.04)	0.07
Platelets	5.21 ± 0.87	6.08 ± 0.87	−0.81 (−1.28, −0.34)	0.001
MNCs	3.94 ± 0.70	4.54 ± 0.70	−0.56 (−1.01, −0.12)	0.01

1 MNC, blood mononuclear cell; PC, phosphatidylcholine; 2C, 2 continuous portions; 2I, 2 intermittent portions.

2 Unadjusted sample mean ± SD for the group at 12 mo is presented.

3 Data are adjusted mean differences (95% CIs) between groups at 12 mo calculated in the mixed-effects model. Mixed-effects models were adjusted for baseline EPA, DHA, or EPA + DHA, age, sex, study center, baseline BMI, and average compliance (percentage).

## Discussion

This study specifically explored whether the pattern and frequency of EPA and DHA intake could alter enrichment of EPA and DHA into plasma fractions and cell membranes in a controlled experimental study in healthy human participants. This was tested by the provision of capsules with the same weekly dose of EPA + DHA given on 2 random days per week only or given as a continuous regimen, divided equally between all days of the week and measuring the enrichment of EPA and DHA into plasma fractions and cell membranes found previously to reflect short-term (plasma phosphatidylcholine), medium-term (platelets), and long-term (MNCs) dietary intake (15).

This study shows that EPA + DHA equivalent to the amount found in 2 portions of oily fish delivered in the continuous regimen for 12 mo leads to a greater enrichment of EPA and EPA + DHA into MNCs and of EPA, DHA, and EPA + DHA into platelets than if the same dose is delivered intermittently on 2 d of the week. However, although there was a trend for a similar finding, this was not statistically significant for EPA, DHA, or EPA + DHA in plasma phosphatidylcholine or for DHA in MNCs in response to the 12-mo intervention.

Extrapolations are made frequently from the findings of n–3 PUFA supplementation studies to public health recommendations for the consumption of oily fish. However, the enrichment of EPA and DHA in response to daily supplements may differ from that in response to the consumption of fish, which is generally sporadic. To the best of our knowledge, this is the first study to simulate sporadic fish consumption in a manner that is directly comparable with the continuous delivery of EPA and DHA in conventional supplementation studies.

There have been studies that directly compared short-term (<8 wk) capsule supplementation with oily fish consumption, in which the oily fish forms part of a meal and replaces other dietary items. Elvevoll et al. (18) compared 2 portions/wk cooked salmon, equivalent to 400 g/wk and providing an average of 1.2 g/d EPA and DHA, to cod liver oil providing 3.0 g/d EPA and DHA. The authors found that the increase in serum EPA and DHA was higher after the intermittent pattern in the cooked salmon group, despite its lower provision of EPA and DHA per day. This is in contrast to our study, in which the continuous pattern resulted in higher EPA and DHA in MNCs and platelets. Visioli et al. (19) reported that plasma EPA and DHA after daily provision of 100 g of salmon was comparable with that after a daily fish oil supplement containing more than double the dose of EPA and DHA. Together, these studies suggest that EPA and DHA, when consumed in fish flesh, may be more bioavailable because it is provided in a form that is more favorable for digestion and absorption of fats than EPA and DHA provided as supplements. The form of the supplements, either TGs (15, 18) or ethyl esters (19), may also play a role in the bioavailability of EPA and DHA.

In keeping with our study design, Harris et al. (20) provided 2 portions of oily fish per week or daily capsules, both of which contained equivalent weekly amounts of EPA and DHA. Their results showed similar proportions of EPA and DHA in plasma phospholipids and RBCs after the 16-wk study. Although this study was smaller than ours (23 participants), it similarly shows no significant difference in the proportion of EPA and DHA in a plasma lipid fraction between the 2 study designs.

In this study, we report a significant difference in the change in reported n–6 PUFA intake between the 2 groups. However, with additional adjustment for this in the statistical model, the results still show the same significant differences in EPA and DHA in platelets and MNCs between the 2 groups. The difference in dietary n–6 PUFA is also evident at baseline, whereas the plasma phosphatidylcholine, platelets, and MNCs results show no difference in EPA and DHA amounts between the 2 groups at baseline. Therefore, we conclude that the differences in dietary n–6 PUFA intake are not responsible for the differences in EPA and DHA between the 2I and 2C groups at 12 mo in this study.

Although there was an overall difference in EPA and DHA incorporation over the course of the 12 mo, we show no significant difference in plasma phosphatidylcholine at 12 mo. It is of interest that a difference between the 2I and 2C groups appears to exist for plasma phosphatidylcholine in the first few weeks of the current study (as illustrated in Fig. 3), but this effect diminished over the course of the 12-mo intervention, and indeed, at 12 mo, there were no significant differences in EPA or DHA between the 2 groups. Rather, it was MNCs and platelets that showed significant differences at 12 mo. We showed previously that plasma fractions respond more acutely to changes in EPA and DHA intake (weeks), whereas platelets and MNCs reflect longer-term changes (15). Compliance in this study was reported to be high (average of >97% across the 12-mo intervention), and the analyses were adjusted for compliance; however, it is possible that, with the duration of the study, compliance to the capsule intervention fell. This would be expected to be most evident in the plasma phosphatidylcholine results, which reflect shorter-term intake. It is also possible that regular intake of EPA and DHA stimulates a more efficient uptake and incorporation of these FAs, as is seen for other nutrients, such as iron (21).

We reported previously similar data for 0, 1, 2, and 4 continuous portions of oily fish (15). Here the adjusted mean difference between the 2I and 2C groups is only marginally smaller than the difference observed between groups receiving doses equivalent to 0 and 1 portion of oily fish per week (range of 0.47–0.94 × the difference, for EPA, DHA, or EPA + DHA in each of plasma phosphatidylcholine, platelets, and MNCs), supporting a focus in dietary guidelines on the total weekly intake of these FAs. However, the difference between the 2I and 2C groups suggests a benefit of frequent consumption and supports weekly rather than monthly food-based guidelines.

The results of this study demonstrate that EPA and DHA enrichment of platelets and MNCs is greater when provided as a continuous daily supply compared with the same amount in “portions” provided intermittently over the course of a 12-mo intervention to mimic oily fish guidelines. This finding may have implications for the associated health benefits observed in continuous supplementation studies and suggests that the same dose of EPA and DHA achieved through sporadic oily fish consumption may have a lesser impact on EPA and DHA status. Additional research to determine the differences in bioavailability and incorporation of n–3 PUFAs derived from oily fish consumption and from supplements and to further explore the consequences of altering cell membrane composition are warranted. In the future, a portions or intermittent study design, such as we used here, may help bridge the gap between experimental studies and public health advice.

## Supplementary Material

Online Supporting Material

## Footnotes

8 Abbreviations used: MNC, blood mononuclear cell; 2C, 2 continuous portions; 2I, 2 intermittent portions.

## References

[1] Kromhout D Feskens EJ Bowles CH The protective effect of a small amount of fish on coronary heart disease mortality in an elderly population. Int J Epidemiol. 1995;24:340–5. 7635594 10.1093/ije/24.2.340

[2] Kromhout D Bosschieter EB de Lezenne Coulander C The inverse relation between fish consumption and 20-year mortality from coronary heart disease. N Engl J Med. 1985;312:1205–9. 3990713 10.1056/NEJM198505093121901

[3] Albert CM Hennekens CH O’Donnell CJ Ajani UA Carey VJ Willett WC Ruskin JN Manson JE Fish consumption and risk of sudden cardiac death. JAMA. 1998;279:23–8. 9424039 10.1001/jama.279.1.23

[4] Hu FB Bronner L Willett WC Stampfer MJ Rexrode KM Albert CM Hunter D Manson JE Fish and omega-3 fatty acid intake and risk of coronary heart disease in women. JAMA. 2002;287:1815–21. 11939867 10.1001/jama.287.14.1815

[5] Atkinson C Whitley E Ness A Baker I Associations between types of dietary fat and fish intake and risk of stroke in the Caerphilly Prospective Study (CaPS). Public Health. 2011;125:345–8. 21636104 10.1016/j.puhe.2011.03.002

[6] Patel PS Sharp SJ Luben RN Khaw KT Bingham SA Wareham NJ Forouhi NG Association between type of dietary fish and seafood intake and the risk of incident type 2 diabetes: the European prospective investigation of cancer (EPIC)-Norfolk cohort study. Diabetes Care. 2009;32:1857–63. 19592633 10.2337/dc09-0116 PMC2752921

[7] Wallin A Di Giuseppe D Orsini N Patel PS Forouhi NG Wolk A Fish consumption, dietary long-chain n–3 fatty acids, and risk of type 2 diabetes: systematic review and meta-analysis of prospective studies. Diabetes Care. 2012;35:918–29. 22442397 10.2337/dc11-1631 PMC3308304

[8] Moore CS Bryant S Mishra GD Krebs JD Browning LM Jebb SA Oily fish reduces plasma triacylglycerols: a primary prevention study in overweight men and women. Nutrition. 2006;22:1012–24. 17027436 10.1016/j.nut.2006.07.005

[9] Miles EA Noakes PS Kremmyda LS Vlachava M Diaper ND Rosenlund G Urwin H Yaqoob P Rossary A Farges MC The Salmon in Pregnancy Study: study design, subject characteristics, maternal fish and marine n–3 fatty acid intake, and marine n–3 fatty acid status in maternal and umbilical cord blood. Am J Clin Nutr. 2011;94:1986S–92S. 21849598 10.3945/ajcn.110.001636

[10] Zhang J Wang C Li L Man Q Meng L Song P Froyland L Du ZY Dietary inclusion of salmon, herring and pompano as oily fish reduces CVD risk markers in dyslipidaemic middle-aged and elderly Chinese women. Br J Nutr. 2012;108:1455–65. 22221492 10.1017/S0007114511006866

[11] Kris-Etherton PM Harris WS Appel LJ Fish consumption, fish oil, omega-3 fatty acids, and cardiovascular disease. Circulation. 2002;106:2747–57. 12438303 10.1161/01.cir.0000038493.65177.94

[12] De Caterina R n–3 fatty acids in cardiovascular disease. N Engl J Med. 2011;364:2439–50. 21696310 10.1056/NEJMra1008153

[13] Harris WS Pottala JV Lacey SM Vasan RS Larson MG Robins SJ Clinical correlates and heritability of erythrocyte eicosapentaenoic and docosahexaenoic acid content in the Framingham Heart Study. Atherosclerosis. 2012;225:425–31. 22727409 10.1016/j.atherosclerosis.2012.05.030 PMC3593234

[14] Scientific Advisory Committee on Nutrition (SACN) and Committee on Toxicity (COT) Combined Report. Advice on fish consumption: benefits and risks. London: Her Majesty’s Stationery Office; 2004.

[15] Browning LM Walker CG Mander AP West AL Madden J Gambell JM Young S Wang L Jebb SA Calder PC Incorporation of eicosapentaenoic and docosahexaenoic acids into lipid pools when given as supplements providing doses equivalent to typical intakes of oily fish. Am J Clin Nutr. 2012;96:748–58. 22932281 10.3945/ajcn.112.041343 PMC3441107

[16] Food Standards Agency. McCance and Widdowson’s the composition of foods. 6th ed Cambridge, UK: Royal Society of Chemistry; 2002.

[17] Food Standards Agency. Food portion sizes. 3rd ed London: Her Majesty’s Stationary Office; 1988.

[18] Elvevoll EO Barstad H Breimo ES Brox J Eilertsen KE Lund T Olsen JO Osterud B Enhanced incorporation of n–3 fatty acids from fish compared with fish oils. Lipids. 2006;41:1109–14. 17269556 10.1007/s11745-006-5060-3

[19] Visioli F Rise P Barassi MC Marangoni F Galli C Dietary intake of fish vs. formulations leads to higher plasma concentrations of n–3 fatty acids. Lipids. 2003;38:415–8. 12848287 10.1007/s11745-003-1077-x

[20] Harris WS Pottala JV Sands SA Jones PG Comparison of the effects of fish and fish-oil capsules on the n 3 fatty acid content of blood cells and plasma phospholipids. Am J Clin Nutr. 2007;86:1621–5. 18065578 10.1093/ajcn/86.5.1621

[21] Mastrogiannaki M Matak P Peyssonnaux C The gut in iron homeostasis: role of HIF-2 under normal and pathological conditions. Blood. 2013;122:885–92. 23678007 10.1182/blood-2012-11-427765 PMC3743464

