# Supplementary material for: Compared with Daily, Weekly n–3 PUFA Intake Affects the Incorporation of Eicosapentaenoic Acid and Docosahexaenoic Acid into Platelets and Mononuclear Cells in Humans
Source: J Nutr. 2014 Mar 19;144(5):667–72. doi: 10.3945/jn.113.186346 (PMC3985823; doi:10.3945/jn.113.186346)
Supplement: Online Supporting Material [file supp_144_5_667__index.html]

Online Supporting Material 

# Compared with Daily, Weekly n–3 PUFA Intake Affects the Incorporation of Eicosapentaenoic Acid and Docosahexaenoic Acid into Platelets and Mononuclear Cells in Humans

## Online Supporting Material

**Files in this Data Supplement:**

- Online Supporting Material - Figure 1
