# Supplementary figures and images for: Compared with Daily, Weekly n–3 PUFA Intake Affects the Incorporation of Eicosapentaenoic Acid and Docosahexaenoic Acid into Platelets and Mononuclear Cells in Humans
Source: J Nutr. 2014 Mar 19;144(5):667–72. doi: 10.3945/jn.113.186346 (PMC3985823; doi:10.3945/jn.113.186346)

Supplemental Figure 1

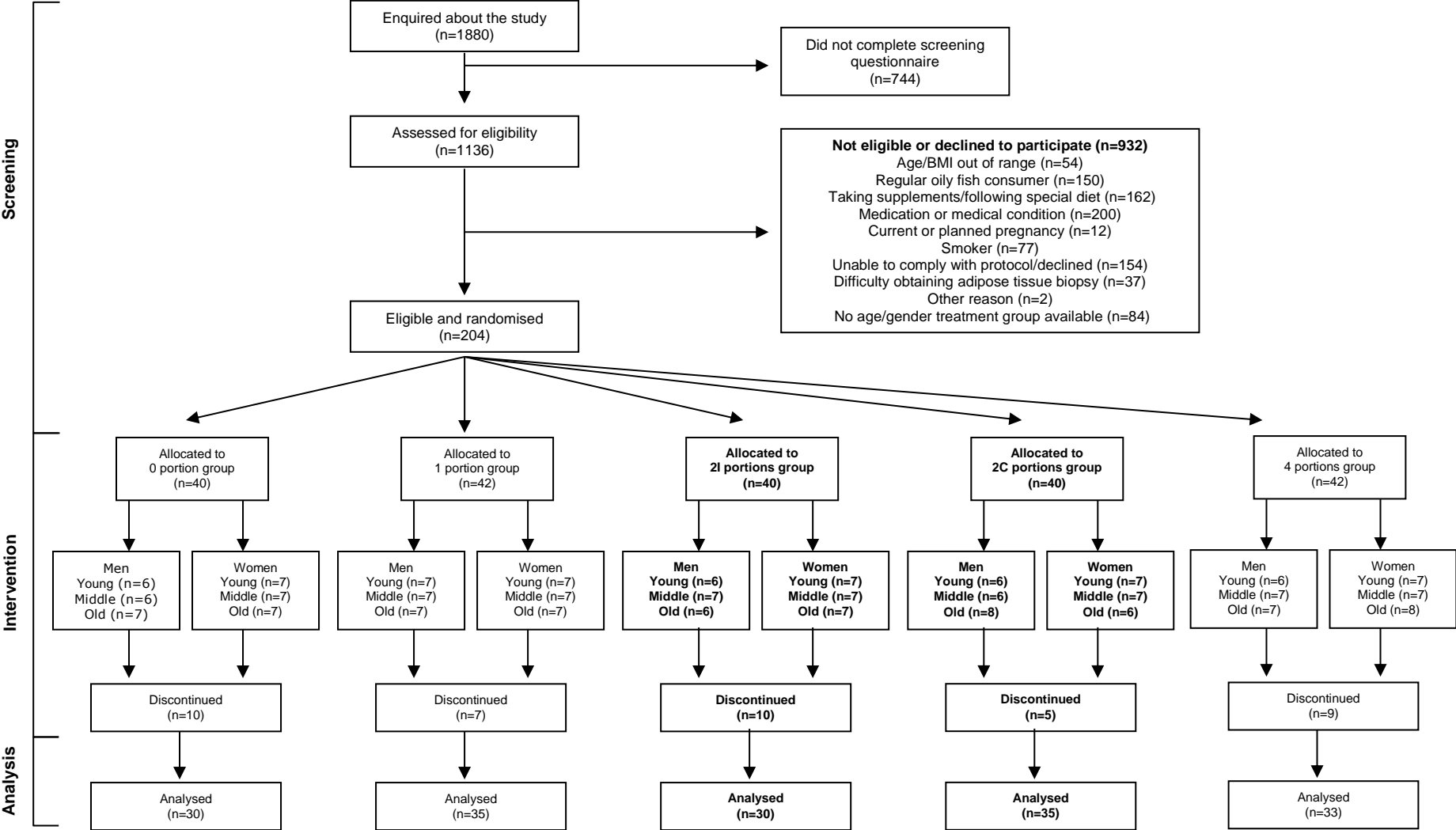

Supplement: Online Supporting Material [file jn.113.186346_nutrition186346SupplementaryData1.pdf]
